# A Case of Hepatic Angiomyolipoma Which Was Misdiagnosed as Hepatocellular Carcinoma in a Hepatitis B Carrier

**DOI:** 10.1155/2012/606108

**Published:** 2012-10-18

**Authors:** Jin Yeon Hwang, Sung Wook Lee, Yang Hyun Baek, Jong Han Kim, Ha Yeon Kim, Suck Hyang Bae, Jin Han Cho, Hee Jin Kwon, Jin Sook Jeong, Young Hoon Roh, Sang Young Han

**Affiliations:** ^1^Department of Internal Medicine, Dong-A University College of Medicine, Busan 602-715, Republic of Korea; ^2^Department of Radiology, Dong-A University College of Medicine, Busan, Republic of Korea; ^3^Department of Pathology, Dong-A University College of Medicine, Busan, Republic of Korea; ^4^Department of Surgery, Dong-A University College of Medicine, Busan, Republic of Korea

## Abstract

We report a rare case of resected hepatic AML, which was misdiagnosed as hepatocellular carcinoma in a chronic hepatitis B carrier. A 45-year-old woman who was a carrier of hepatitis B virus infection presented with a hepatic tumor. Her serum alpha-fetoprotein level was normal. Ultrasonography revealed a round and well-circumscribed echogenic hepatic tumor measuring 2.5 cm in the segment VI. On contrast-enhanced computed tomography, a hypervascular tumor was observed in the arterial phase and washing-out of the contrast medium in the portal phase and delayed phase. On MR T1-weighted in-phase images, the mass showed low signal intensity, and on out-of-phase images, the mass showed signal drop and dark signal intensity. On MR T2-weighted images, the mass showed high signal intensity. The mass demonstrated high signal intensity on arterial phase after contrast injection, suggestive of hepatocellular carcinoma. The patient underwent hepatic wedge resection and histopathological diagnosis was a hepatic angiomyolipoma.

## 1. Introduction

Angiomyolipoma (AML) typically occurs in the kidney and rarely in liver [1]. Hepatic AML is a rare, primarily benign mesenchymal tumor, composed of blood vessels, fat tissue, and smooth muscle cells [2]. Ishak reported the first hepatic AML in 1976 [3] and since then, there have been about 200 cases reported in the literature and they have been increasing with improvement in imaging modalities, including ultrasonography (US), computed tomography (CT), magnetic resonance imaging (MRI), and fine-needle aspiration biopsy (FNAB) [4]. The hepatic AML may pose a diagnostic challenge clinically, radiologically, and pathologically because of its wide variation due to the different proportions of the three cell types which make up the tumor. In particular, in a region endemic for hepatocellular carcinoma, the diagnosis of AML by imaging modality can be difficult and frequently misdiagnosed as hepatocellular carcinoma. The definitive diagnostic study remains the histological examination coupled with immunohistochemical stains. Among the components of hepatic AML, homatropine methyl bromide 45 (HMB-45) positive smooth muscle cell is the only specific and definitive criterion for diagnosis [5]. Hepatocellular carcinoma and liver hemangioma are negative for this marker. We report a case of resected hepatic AML, which was misdiagnosed as hepatocellular carcinoma in a hepatitis B carrier.

## 2. Case Report

A 45-year-old woman, a chronic hepatitis B carrier, was admitted to our hospital for further evaluation and treatment of a liver mass that had been found on abdominal US at regular medical checkup in November 11, 2011. Clinically, no pathologic findings were observed during physical examination. All routine blood investigations, including liver function tests, were normal. The serologic studies for viral hepatitis B showed only positive hepatitis B surface antigen (HBs Ag) test result. The serologic markers for hepatitis C were nonreactive. The hepatitis B virus DNA titer was 438 IU/mL (1,495 copies/mL) and serum alpha-fetoprotein level was 2.01 ng/mL (normal <20 ng/mL).

**Figure 1:**
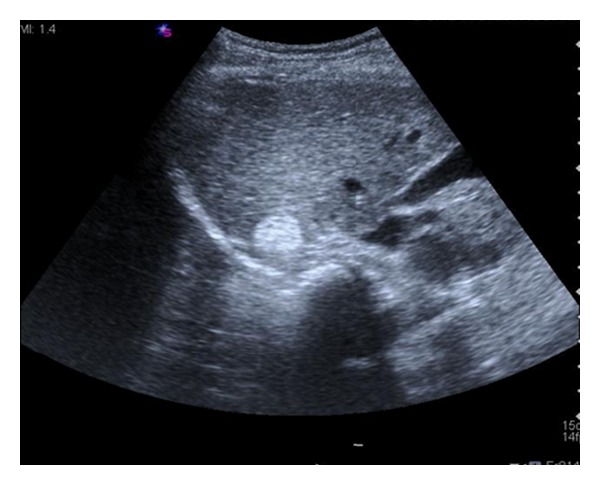
Ultrasonographic findings of the liver mass. Ultrasonogram demonstrates a 2.5 cm sized round, well-marginating hyperechoic mass.

**Figure 2:**
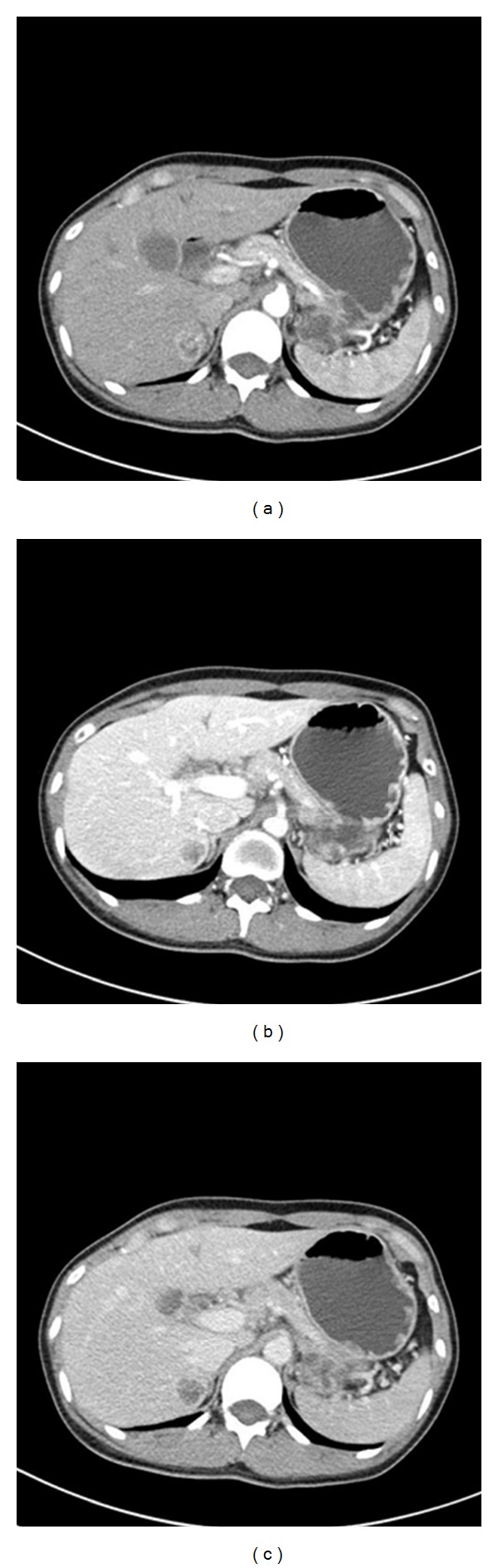
CT findings of the liver mass. Contrast-enhanced CT revealed a heterogeneous hypervascular mass in the arterial phase (a) and washing-out of the medium in the portal (b) and delayed phases (c).

**Figure 3:**
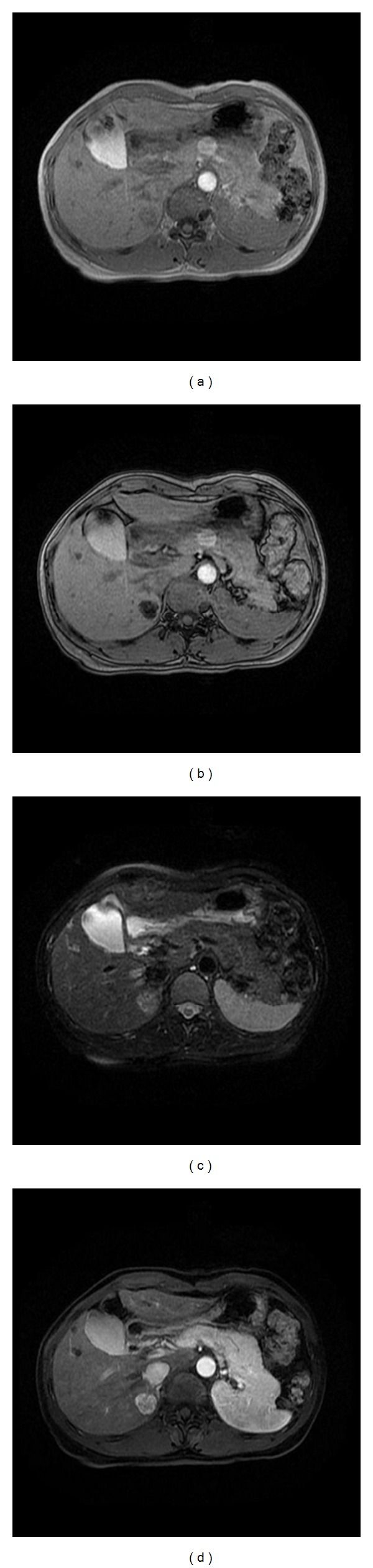
MR findings of the liver mass. On MR T1-weighted in-phase images, the mass shows low signal intensity (a), and on out-of-phase images, the mass shows signal drop and dark signal intensity (b). On MR T2-weighted images, the mass shows high signal intensity (c). The mass demonstrates high signal intensity on arterial phase after contrast injection (d).

Abdominal US showed a well-defined, hyperechoic mass, with maximal diameter of 2.5 cm in the segment VI of the liver (Figure 1). On contrast-enhanced computed tomography (CT), a hypervascular tumor was observed in the arterial phase (Figure 2(a)) and washing-out of the contrast medium in the portal phase and delayed phase (Figures 2(b) and 2(c)). On abdominal magnetic resonance (MR) images, the lesion showed low signal intensity on the T1-weighted in-phase images (Figure 3(a)) and showed signal drop and dark signal intensity on the T1-weighted out-of-phase images (Figure 3(b)). On MR T2-weighted images, the mass shows high signal intensity (Figure 3(c)). The mass demonstrates high signal intensity on arterial phase after contrast injection (Figure 3(d)), suspicious for fat containing hepatocellular carcinoma.

**Figure 4:**
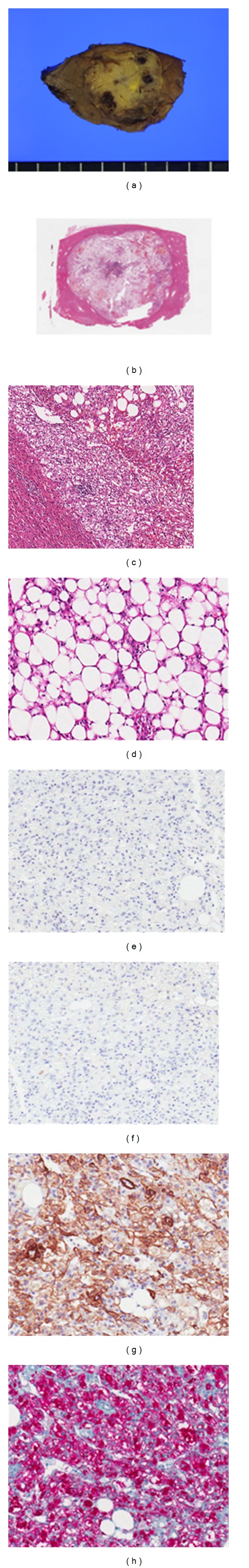
Pathologic findings of the liver mass ((a) Gross, (b)–(d); Hematoxylin & eosin stain, (e)-(f); Immnuohistochemistry ((e) anti-CK8/18, (f) anti-HSA, (g) anti-SMA, (h) anti-HMB45, (e)–(g) brown chromogen, (h) red chromogen), (b) ×1, (c) and (d) ×40, (e)–(h) ×200). The liver shows a well-demarcated mass with yellowish cut surface and hemorrhage. Microscopically, the mass composes of fat cells, vessels and epithelioid cells. The epithelioid cells exhibit non-epithelial origin (negative CK8/18 and negative HSA), SMA (+) and HMB45 (+).

As hepatocellular carcinoma was highly suspected from preoperative image studies with her medical history, hepatic wedge-resection with tumor in the segment VI was performed. Wedge resected liver showed a well-demarcated but nonencapsulated soft mass, representing variegated cut surface with yellowish fat tissue, multifocal hemorrhage, and no necrosis (Figure 4(a)). The mass composed of blood vessels, fat tissue and areas of epithelioid cells, and some inflammatory cells (Figures 4(b)–4(d)). The epithelioid cells showed monotonous round nuclei and clear cytoplasm and anti-CK8/18 (−) and anti-HSA (−), which were recognized as nonliver cells (Figures 2(e), and 2(f)). The epithelioid cells were anti-smooth muscle antibody (SMA) (+) (Figure 4(g)), anti-vimentin (+), and anti-S100 (−), representing smooth muscle cell. Furthermore, the epithelioid cells show strong anti-HMB45 (+) (Figure 4(h)), which has been known as a unique marker for AML. Pathologically, the tumor was diagnosed as an AML of the liver, benign. The patient recovered uneventfully and was discharged 1 week after operation.

## 3. Discussion

Angiomyolipoma (AML) is an uncommon mesenchymal tumor that occurred more frequently in kidney than in liver [1]. Most of the patients have no symptoms or signs; the majorities were found incidentally on routine medical examination using ultrasound. Preoperative diagnosis of hepatic AML mostly relies on imaging studies and the radiological characteristics of the lesion have been described in some of the reported cases [2, 6, 7]. It is typically echogenic on ultrasound, hypodense on precontrast CT scans, markedly enhanced on arterial phase, and remained in enhancement with portal venous phase. MR imaging characteristics vary depending on the proportion of intratumoral fat [6]. Commonly, AML has a high fat content, with high signal intensity on T1-weighted images and a significant drop in signal intensity on fat-suppressed images. However, the imaging feature of hepatic AML varies because of variations in the proportion of adipose cells, smooth muscle cells, and vessels. In particular, the number of adipose cells varies between 10% and 90% [7]. This heterogeneity makes the preoperative diagnosis by imaging quite difficult, and it is possible to misdiagnose hepatic AML as a number of entities, both benign and malignant [7–13]. Commonly confused entities include lipoma, hepatocellular adenoma, hepatocellular carcinoma with fatty metamorphosis, sarcoma, or other metastatic neoplasm. Notably, hepatic AML has been misdiagnosed as hepatocellular carcinoma with a frequency more than 50% due to a significant overlap of the imaging features [8, 9, 13]. Some studies place emphasis on the differentiation of hepatic AML and fat-containing hepatocellular carcinoma that usually arise from the cirrhotic liver [8, 10].

The definitive diagnostic study remains the histological examination coupled with immunohistochemical stains. Among the components of hepatic AML, HMB-45 positive smooth muscle cell is the only specific and definitive criterions for diagnosis [5]. Hepatocellular carcinoma and liver hemangioma are negative for this marker.

In the present case, the preoperative radiological image was quite difficult to distinguish from fat-containing hepatocellular carcinoma. Although her serum alpha-fetoprotein was normal, the radiological findings and her clinical history are compatible with hepatocellular carcinoma. Under the above impression, these lesions were resected and the histological examination coupled with immunohistochemical stains made the final diagnosis of hepatic AML. Because the preoperative images showed atypical findings for AML, we did not make a accurate diagnosis of hepatic AML at that time.

The management of hepatic AML sometimes remains controversial. Several authors have suggested that this disease can be managed with conservative treatment with followup after fine-needle aspiration biopsy in previous series [1, 14, 15]. Some reports recommended that surgical intervention may be needed in selected cases to alleviate the mass effect on the neighboring organs [1, 16], and very few cases of AML with concomitant hepatocellular carcinoma, malignant transformation, and its spontaneous rupture have been reported [16–20]. No surgical treatment of AML in an endemic area for hepatocellular carcinoma should proceed with caution because cases of fat-contained hepatocellular carcinoma will make the diagnosis difficult. Given the high prevalence of hepatocellular carcinoma in Korea, the decision for surgical intervention is straightforward if imaging and laboratory studies are equivocal. 

In conclusion, preoperative diagnosis of hepatic AML by image is sometimes quite difficult particularly in endemic areas of hepatocellular carcinoma, and in the patients who have risk factors of hepatocellular carcinoma with suggested malignancy by image but showing normal laboratory findings, repeated studies with different diagnostic modalities, such as biopsy or angiography, and careful interpretation are recommended.
